# Resveratrol Down-Regulates Myosin Light Chain Kinase, Induces Apoptosis and Inhibits Diethylnitrosamine-Induced Liver Tumorigenesis in Rats

**DOI:** 10.3390/ijms14011940

**Published:** 2013-01-17

**Authors:** Xiao-Lin Zhang, Hao Yu, You-Yi Xiong, Shi-Tang Ma, Lei Zhao, Shi-Feng She

**Affiliations:** 1 Food and Drug College of Anhui Science and Technology University, Bengbu 233100, Anhui, China; E-Mails: yhz_1230@163.com (H.Y.); xyytc1@163.com (Y.-Y.X.); nothingchina@126.com (S.-T.M.); 2 Animal Science College of Anhui Science and Technology University, Bengbu 233100, Anhui, China; E-Mail: zhaolei918673@yahoo.com.cn; 3 Department of Digestive Medicine, The First Affiliated Hospital of Guangzhou University of Chinese Medicine, Guangzhou 510405, Guangdong, China

**Keywords:** myosin light chain kinase, liver tumorigenesis, resveratrol, apoptosis

## Abstract

Hepatocellular carcinoma (HCC) is a serious healthcare problem worldwide because of its increasing morbidity and high mortality rates. However, our understanding of the mechanism of liver tumorigenesis remains incomplete. We report the expression of myosin light chain kinase (MLCK) in the livers of rats with diethylnitrosamine (DENA)-induced HCC and investigated the correlation between MLCK and liver tumorigenesis by observing the expression of MLCK in a rat model of HCC. HCC was induced in rats by an intraperitoneal injection of DENA, and resveratrol-treated rats were orally administered resveratrol with 50 mg/kg body weight/day. The livers of rats were excised after 20 weeks and immersed in 10% formaldehyde prior to immunohistochemical and Western blot analyses for determining the level of MLCK expression. These analyses indicated that the MLCK expression was higher in the livers of HCC rats than in normal and resveratrol-treated rats. High level of MLCK expression was responsible for proliferation and anti-apoptotic effects. However, resveratrol down-regulated the expression of MLCK, which induced cell apoptosis and inhibited liver tumorigenesis in rats with DENA-induced HCC. Our results suggest that the over expression of MLCK may be related to the development of liver tumorigenesis.

## 1. Introduction

Hepatocellular carcinoma (HCC), the most common type of liver cancer, is the fifth most common malignant tumor type worldwide and the second leading cause of cancer-related death [1,2]. Chronic inflammation of the liver and subsequent cirrhosis are highly correlated with hepatitis B and hepatitis C viral infections and alcoholic and metabolic liver diseases [2,3]. Additionally, obesity, environmental pollutants, aflatoxin infection and nitrosamine consumption are the strongest risk factors for HCC development [4–6]. The highest incidences of HCC occur in sub-Saharan Africa and southeastern Asia, where hepatitis B virus infection is endemic [7]. The prognosis of HCC is poor, and the overall five-year survival rate worldwide is estimated at only 3%, mainly because HCC is frequently diagnosed at an advanced stage. Currently, there is no proven effective systemic chemotherapy for HCC. In light of the limited treatment options and grave prognosis, chemoprevention has been considered to be the best strategy to lower the morbidity and mortality rates associated with liver cancer [8].

Resveratrol, a phytoalexin that is readily available from several dietary sources, is reported to possess antitumorigenic properties in several cancers [9], and it has been shown to suppress the proliferation of a wide variety of human tumor cells *in vitro* [10]. Studies have shown that resveratrol can prevent or slow the progression of a wide variety of illnesses, including cancer [11,12]. Experimental findings have revealed multiple cellular targets of resveratrol that affect cellular proliferation and growth, apoptosis, inflammation, invasion, angiogenesis and metastasis [13]. There have been reports that resveratrol significantly prevents diethylnitrosamine (DENA)-induced hepatic tumorigenesis by mechanisms such as antioxidant, anti-inflammatory and anti-angiogenic effects, and alteration of hepatic proinflammatory cytokines in rats [14–18]. However, the underlying mechanisms of the inhibitory effects of this dietary polyphenol against rat liver carcinogenesis still need to be studied to further understand this process from other points of view.

Apoptosis, also called programmed cell death, is a physiological cell suicide program in eukaryotic cells that has been suggested to play an important role in the maintenance of homeostasis by facilitating natural tissue turnover [19,20]. Moreover, interactions between cells and extracellular matrix (ECM) also play an important role in development and normal cellular function. Cell adhesion to ECM is a key factor in cellular homeostasis, and the disruption of such interactions leads to a specific type of apoptosis known as “anoikis” in most non-transformed cell types. Apoptosis following the loss of cell anchorage influences development, tissue homeostasis and disease. Anchorage-independent growth is a crucial step during tumorigenesis, and there is increasing evidence indicating that the inhibition of anoikis enhances adhesion signaling in cell-ECM contact sites in tumor cells [21]. Integrins, which are heterodimeric membrane glycoproteins, are a family of cell-adhesion receptors that mediate cell-cell and cell-ECM interactions. The analysis of tumor-associated integrins has revealed an important relationship between integrins and tumorigenesis [22]. Integrins can sense mechanical forces arising from the matrix and convert these stimuli to chemical signals that are capable of modulating intracellular signal transduction. Integrin-mediated cell migration is a crucial step during tumorigenesis and, in particular, during the metastatic spreading of cancer cells. Integrin-mediated cell migration requires the contractile forces generated by the actin cytoskeleton that are mediated by the actin/myosin network through integrin-ECM interactions. Actin filaments are cross-linked by myosin complexes; this cross-linkage results in the bundling and contraction of the actin fibers [23]. The contractile forces are regulated by myosin light chain (MLC) phosphorylation via MLC kinase (MLCK). MLCK is a Ca^2+^/calmodulin-dependent protein kinase that regulates a variety of cellular functions, such as muscle contraction and cell migration. A previous study identified a direct correlation between the levels of MLCK expression and the reoccurrence of non-small cell lung cancer [24]. Additional studies have shown that MLCK is involved in other key aspects of tumorigenesis, including the growth of primary tumors and tumor cell motility in human pancreatic cancer cells and Mm5MT mouse mammary tumor cells [25,26]. However, few studies have investigated the association between MLCK and HCC. In this paper, the relationship between MLCK and HCC is described, the variable expression of MLCK during the development of HCC and the effects of resveratrol on DENA-induced hepatocarcinogensis, apoptosis and MLCK expression are investigated.

## 2. Results and Discussion

### 2.1. Body and Liver Weights and Nodule Growth

Rats were subjected to the experimental conditions described in the Experimental Section. The experiment was terminated 17 weeks after the final carcinogen dose. The final body weights of the rats in the carcinogen (DENA) control group (Group C) and the DENA+ carboxymethylcellulose-treated group (Group D) groups were slightly lower than those of rats in the normal control group (Group A) and carboxymethylcellulose-treated group (Group B) (data not statistically significant). Treatment with resveratrol (50 mg/kg/day) increased the final body weights of the animals in Group E compared to those in Groups C and D (data not statistically significant). There were no statistically significant differences in the liver weights between the groups. The relative liver/body weights of the rats in Groups C and D were found to be significantly higher (*p* < 0.01) than those of the rats in Groups A and B (Table 1).

**Figure 1:**
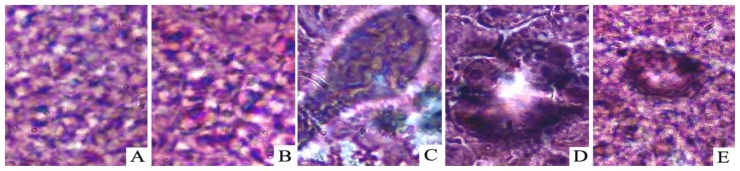
Hematoxylin/Eosin (H & E) staining of the non-nodular liver sections (400×). Normal control (**A**) and carboxymethylcellulose-treated (**B**) rat livers showing normal cellular architecture. Images of DENA control (**C**) rat liver and DENA+ carboxyme-thylcellulose-treated (**D**) rat liver tissues showing areas of aberrant hepatocellular phenotypes with variations in nuclear size, hyperchromatism, and irregular sinusoids. Liver section from DENA + resveratrol-treated (50 mg/kg body weight/day) (**E**) rat liver showing obvious improvement of hepatic histology compared with those of Groups C and D.

There were no visible hepatocyte nodules in the livers of rats in the normal control (Group A, *n* = 8) and carboxymethylcellulose-treated (Group B, *n* = 8) groups. Table 2 summarizes the incidence of nodules, the total number of nodules and the average number of nodules per nodule-bearing liver of the DENA-treated groups with or without resveratrol treatment. A significantly decreased incidence of nodules was observed in the group that received the resveratrol treatment throughout the experiment (Group E, 5/8 = 62.5%) relative to the DENA-treated (Group C, 8/8 = 100%) and DENA + carboxymethylcellulose-treated (Group D, 8/8 = 100%) groups (*p* < 0.05). Similarly, the average number of nodules per nodule-bearing liver (nodule multiplicity) was smaller in Group E (10.1 ± 2.6) than in Groups C (36.8 ± 4.3) and D (36.2 ± 6.9) (*p* < 0.01). A histopathological examination of the liver non-nodular tissues confirmed the protective effect of resveratrol (Figure 1).

### 2.2. Immunohistochemical Examination and Western Blot Analysis

**Figure 2:**
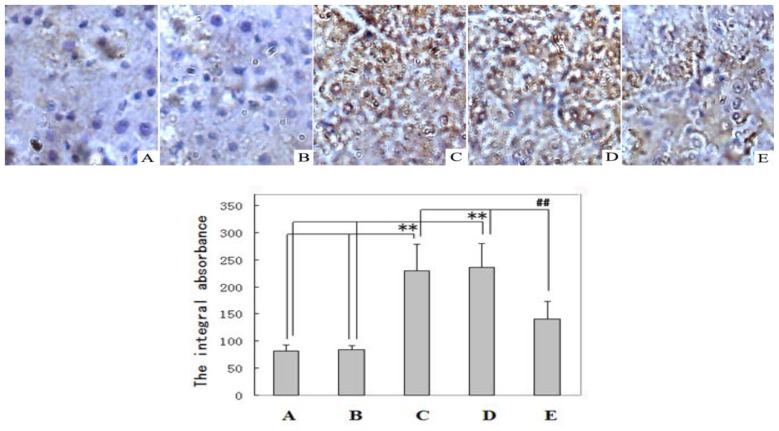
Immunohistochemistry analysis of myosin light chain kinase (MLCK) was performed in non-nodular livers of rats (400×). Normal control (**A**) rat liver and carboxymethylcellulose-treated (**B**) rat liver showing very little MLCK staining. MLCK staining in the DENA control (**C**) rat liver and DENA + carboxymethylcellulose-treated (**D**) rat liver showing brown staining. Liver section from a DENA + resveratrol-treated (**E**) rat liver showing less frequent immunostaining for MLCK-positive foci compared to the liver sections from Groups C and D. ** *p* < 0.01 Groups C and D compared with normal control Group A and Group B, ## *p* < 0.01 Group E compared with DENA control Group C and Group D.

The immunohistochemical examination of the liver tissues for MLCK confirmed that the expression of MLCK and the value of the MLCK integral absorbance in the livers in the DENA control (Group C, 230 ± 48) and DENA + carboxymethylcellulose-treated (Group D, 236 ± 43) rats were higher than in the normal control (Group A, 81 ± 12) and carboxymethylcellulose-treated (Group B, 84 ± 8) animals (*p* < 0.01). The MLCK expression level in the livers of rats receiving resveratrol chemoprevention (Group E, 141 ± 32) was much lower than the MLCK expression levels in the DENA control (Group C, 230 ± 48) and DENA + carboxymethylcellulose-treated (Group D, 236 ± 43) groups (*p* < 0.01) (Figure 2).

**Figure 3:**
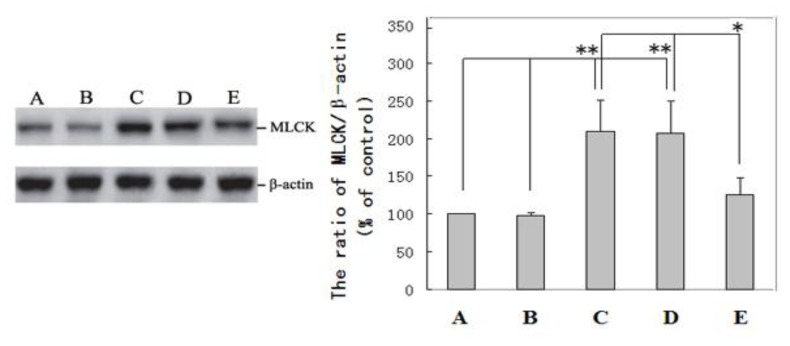
A Western blot analysis of MLCK expression was performed in rat non-nodular livers, and the ratio of MLCK/β-actin was determined (mean ± S.D.). Liver homogenates were prepared as described in the Experimental Section. The Western blot analysis was performed using an anti-MLCK monoclonal antibody and a β-actin polyclonal antibody. Laser scanning densitometry was conducted to quantify the differences. Normal control (Group A). The densitometric units of the control group (MLCK/β-actin) were set to 100, and the internal β-actin control is shown for the carboxymethylcellulose-treated group (Group B), the DENA control group (Group C), the DENA + carboxymethyl-cellulosetreated group (Group D), and the DENA + resveratrol-treated group (Group E). ** *p* < 0.01 Groups C and D compared with normal control Group A and Group B; * *p* < 0.05 Group E compared with DENA control Group C and Group D.

The Western blot examination of the MLCK expression levels in liver tissues confirmed that the expression of MLCK and the ratio of MLCK/β-actin (the internal control) in the livers of the DENA control (Group C, 210 ± 41) and DENA + carboxymethylcellulose-treated (Group D, 207 ± 43) rats were higher than the expression levels in the normal control (Group A, MLCK/β-actin ratio was set to 100) and carboxymethylcellulose (Group B, 98 ± 3) groups (*p* < 0.01). The MLCK expression level in the livers of rats receiving resveratrol chemoprevention (Group E, 125 ± 23) was slightly higher than those in Groups A and B (*p* < 0.05) but much lower than those in Groups C and D (*p* < 0.01). The data were normalized to the internal β-actin control by semiquantitative densitometric scanning (Figure 3).

### 2.3. TUNEL Analysis

**Figure 4:**
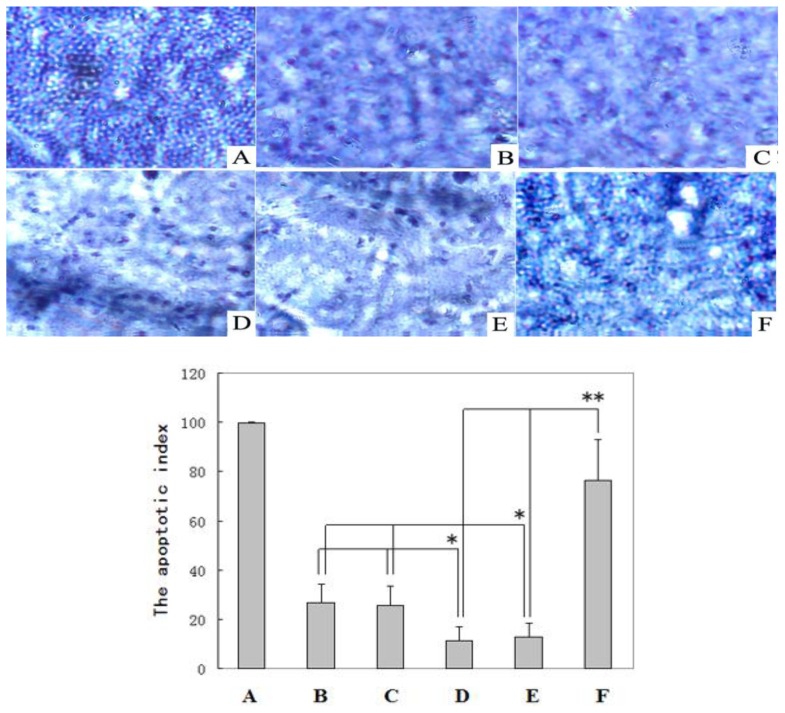
Terminal transferase dUTP nick end labeling (TUNEL) analysis of the apoptotic cells was performed in rat non-nodular liver and positive control slices and the apoptotic index of the rat non-nodular liver cells was determined (mean ± S.D.). (**A**) Positive control (slice obtained from kit, 100%); (**B**) normal control Group A; (**C**) carboxymethylcellulosetreated Group B; (**D**) DENA control Group C, (E) DENA+carboxymethylcellulose-treated Group D; and (**F**) DENA + resveratrol-treated Group E. * *p* < 0.05 Groups C and D compared with normal control Group A and Group B; ** *p* < 0.01 Group E compared with DENA control Group C and Group D.

The apoptosis in the tumor samples from the rats was evaluated *in situ* using the terminal transferase dUTP nick end labeling (TUNEL) methodology as described in the Experimental Section. As shown in Figure 4, a greater number of apoptotic cells was found in tumors from the DENA + resveratrol-treated rats.

The mean percentage of the number of apoptotic cells relative to the number of total cells (the apoptotic index, AI) in the livers of the DENA + resveratrol-treated (Group E) rats (76.4 ± 16.5) was much larger than in the normal control (Group A) rats (26.7 ± 7.6) and carboxymethylcellulose-treated (Group B) rats (25.6 ± 7.8) (*p* < 0.01). The AI of the positive control cells was nearly 100%. Apoptotic cells were rarely detected in the livers of the DENA control (Group C) rats (11.3 ± 5.8) and DENA + carboxymethyl-cellulose-treated (Group D) rats (12.8 ± 5.6). The apoptotic indices (AI) in the livers from Groups C and D rat livers were slightly lower than those in the rat livers from Groups A and B (*p* < 0.05) and considerably lower than that in the rat livers from Group E (*p* < 0.01) (Figure 4).

Over the past few decades, our increased understanding of the carcinogenic process at the cellular and molecular levels has led to the development of a promising new approach to cancer prevention, which is termed “chemoprevention” [27]. This approach aims to halt or reverse the development and progression of pre-cancerous cells by administering non-cytotoxic nutrients and/or pharmacological agents during the time period between tumor initiation and malignancy [28]. Carcinogenesis can be regarded as an accumulation of genetic or biochemical cell damage, and this process could potentially be interrupted at various steps during the initiation, promotion or progression stages [29,30]. Resveratrol has been used in numerous preclinical animal studies to evaluate the chemopreventative and chemotherapeutic potential of this compound with regard to cancer [31–33]. In this study, we confirmed that resveratrol could prevent or delay liver tumorigenesis (Table 1 and Figure 1).

Regulating apoptosis has been reported to be a new strategy for the treatment and prevention of cancer [19,34]. To our knowledge, this study provides the first *in vivo* evidence that the over expression of MLCK is associated with the initiation of HCC by reducing tumor cell apoptosis and that the inhibition of MLCK by resveratrol induces apoptosis and prevents liver tumorigenesis (Figures 2, 3 and 4). Our results are supported by those of the studies, which demonstrate that the inhibition of MLCK activity induces apoptosis *in vivo* [26]. This study also demonstrates that MLCK plays an essential role in the survival of transformed HCC cells. Petrache *et al*. [35], reported that the caspase-dependent cleavage of MLCK plays a role in apoptosis. MLCK might be implicated in the formation of integrin-positive adhesive structures; however, further studies are required to determine how these kinases mediate integrin-dependent functions. In conclusion, resveratrol treatment modulates the levels of MLCK proteins in rat livers, which promotes apoptosis in DENA-induced liver tumors. Our study has clearly shown that resveratrol down-regulates the expression of MLCK, therefore, resveratrol could be useful for the prevention of HCC. The data from this study showed the following: (i) The level of MLCK expression was increased during the DENA-induced initiation of hepatic carcinogenesis; and (ii) resveratrol supplementation prevented the DENA-induced initiation of liver cancer by reducing high MLCK expression to normal levels and inducing tumor cell apoptosis. The latter result indicates the potential applications of resveratrol in preventing, slowing or reversing the liver carcinogenesis. Based on the outcomes of this study, we suggest that resveratrol could be developed as an agent for the prevention of HCC. However, further studies are needed to establish a cause-and-effect relationship between MLCK and the action of resveratrol. The results from this study suggested that carboxymethylcellulose supplementation did not exert an adverse effect on the growth of the rats (Table 1), which confirmed that carboxymethylcellulose is nontoxic in rats. Other results demonstrated that carboxymethylcellulose treatment did not reverse MLCK expression, induce apoptosis or prevent liver carcinogenesis in rats with DENA-induced HCC. However, carboxymethylcellulose could be used as a resveratrol cosolvent.

## 3. Experimental Section

### 3.1. Animals and Chemicals

Male Sprague-Dawley rats, which weighed 65 to 85 g at the beginning of the study, were obtained from Changlinhe Pharmaceutic Co., Ltd., Anhui, China. The animals were housed in polycarbonate cages and fed a commercial pellet diet with water *ad libitum*. All procedures were conducted in accordance with the China legislation No. 8910M047 on the use and care of laboratory animals. The guidelines were established by the Institute for Experimental Animals of Anhui Science and Technology University and were approved by the university committee for animal experiments. DENA was purchased from Sigma Chemical Co., Beijing, China. Resveratrol (98%) was supplied by Ciyuan Pharmaceutical Co., Xian, China and the test of purity was performed and determined using standard and high performance liquid chromatography.

### 3.2. Experimental Design

The rats were randomized into five groups. The animals were acclimatized to the laboratory conditions for approximately seven days before beginning the experiments. The rats in Group A were the normal control animals, while the animals in Group B received 100 mL/kg body weight/day carboxymethylcellulose (10 g/L) orally. Hepatocarcinogenesis was initiated in all other animals by intraperitoneal injection of DENA (100 mg/kg body weight/week) once a week for a period of 3 weeks. The rats were then randomly divided into three groups: A model group (DENA group, Group C), a control group (DENA+carboxymethylcellulose group, Group D) and a resveratrol-treated group (DENA+resveratrol group, Group E). The rats in the resveratrol-treated group (Group E) were given 50 mg/kg body weight/d resveratrol suspended in 100 mL/kg body weight/day carboxymethylcellulose (10 g/L) oral administration of which was done by gavage (*per os*). Due to its low solubility in water, resveratrol was suspended in 10 g/L carboxymethylcellulose at a constant volume of 100 mL/kg body weight before each administration. The rats in the control group (Group D) were given 100 mL/kg body weight/day carboxymethylcellulose (10 g/L) during the same period. All the rats in the model group (Group C) were given tap water with free access to the feed until the experimental rats achieved their endpoints. The doses of resveratrol were calculated based on the consumption of a constant total liquid volume of resveratrol solution equivalent to 50 mg/kg body weight/day. Dose preparation and administration were performed in dim light using opaque plastic ware to avoid exposure of the agent to light. Resveratrol treatment was started 4 weeks before the hepatocarcinogenesis induction and continued for 20 weeks. All rats were sacrificed after 20 weeks.

### 3.3. Tissue Collection and Isolation

The rats in each group (*n* = 8) were sacrificed under anesthesia induced with 3% pentobarbitalsodium sodium. The livers were then resected and subjected to morphologic analysis of the visible hepatocyte nodules according to the methods described by Bishayee *et al.* [36]. A portion of non-nodular liver tissue from each of the different groups was collected, fixed, embedded in paraffin and sectioned for immunohistochemical staining and *in situ* analysis of cell apoptosis. The remaining tissue was cut into thin slices and homogenized or stored at −80 °C for Western blot analysis.

### 3.4. Immunohistochemistry

The liver tissue were sectioned at an average thickness of 5 μm and stored at −20 °C until use. The sections were blocked with 5% BSA (Sigma) and 5% normal rabbit serum (Vector Laboratories, Burlingame, CA, USA) and incubated with the rabbit anti-rat monoclonal MLCK antibody (Epitomics, CA, USA) overnight. The sections were then incubated with HRP-conjugated goat anti-rabbit IgG (1:200 dilution) and treated with a metal-enhanced 3,3′-diaminobenzidine (DAB; Thermo Scientific, Rockford, IL, USA) for 3 min. After thoroughly washing the sections, they were mounted on glass slides with Vectashield (Vector Laboratories, Burlingame, CA, USA). Each section and its integral absorbance of MLCK expression were examined by microscope.

### 3.5. Western Blot Analysis

The liver tissues were washed three times with phosphate buffered saline (PBS) and then lysed in RIPA protein lysis buffer containing a protease inhibitor cocktail (1% Nonidet P-40, 1% sodium deoxycholate, 0.1% SDS, 150 mM NaCl, 10 mM sodium phosphate buffer pH 7.2, 2 mM EDTA, 10 mg/mL aprotinin, 10 mg/mL leupeptin, 2 mM PMSF, 2 mM sodium orthovanadate, 10 mM sodium pyrophosphate, and 20 mM sodium fluoride). The lysates were centrifuged at 15,000× *g* for 30 min at 4 °C, and the supernatants were analyzed by Western blot. The total protein concentration of each sample was determined using the MicroBCA Protein Assay Reagent Kit (Pierce, Rockford, IL, USA). A total of 30 μg of lysate from each sample was incubated at 98 °C for 5 min, separated in a 10% sodium dodecyl sulfate (SDS)-polyacrylamide gel at 120 V for 2 h in 1× running buffer (25 mmol/L Tris, 192 mmol/L glycine and 0.1% SDS (pH 8.3)), and electrophoretically transferred to polyvinylidene difluoride (PVDF) membranes at 95 V for 35 min in 2× tris-glycine transfer buffer with 0.025% SDS. The PVDF membranes were then blocked with 5% fat-free milk in PBST (PBS, 0.1% Tween 20) for 1 h at room temperature. The membranes were incubated with rabbit monoclonal anti-rat MLCK antibody (1:1000 dilution) and rabbit polyclonal anti-rat β-actin antibody (Abcam, CA, UK; 1:500 dilution) for 2 h at room temperature. The membranes were subsequently incubated with an horseradish peroxidase (HRP)-conjugated goat anti-rabbit IgG secondary antibody (1:2000 dilution) and stained with enhanced chemiluminescence reagent (Pierce, Rockford, IL, USA). After exposure to X-ray film, the protein bands were semiquantitatively measured by densitometric scanning. Three independent experiments were performed, and the results were reproducible. The results were expressed as the mean ± S.D. of three replicates for each treatment. Statistical analysis of variance with regard to β-actin, a loading control, was performed, and an optical density variation of at least 5% was required for differences between treatments to be deemed significant.

### 3.6. Detection of in situ Cell Apoptosis by TUNEL Assay

The TUNEL (terminal transferase dUTP nick end labeling) assay was performed to determine the effect of resveratrol on apoptosis using the POD (peroxidase) *in situ* cell death detection kit (Boster, Wuhan, China) according to the manufacturer’s instructions; detection was performed using POD and diaminobenzidine (DAB) substrate. Positive control slides were included in the kit. The sections were counterstained with hematoxylin. The number of apoptotic cells in the tissues of control and resveratrol-treated rats was determined by counting the stained cells in 12 fields, each of which contained at least 50 cells. The cells were visualized with a light microscope. The apoptotic index (AI) was calculated as follows: AI = (number of apoptotic cells/total number of cells) × 100%.

### 3.7. Statistical Analysis

The primary analysis was a comparison among control group, carboxymethylcellulose-treated group, DENA control group, DENA + carboxymethylcellulose-treated group, DENA + resveratrol-treated group. The data of the nodule incidence data was analyzed using Fisher’s exact probability test. Other data were presented as mean ± S.D. With statistical analyses by *t*-test, differences between groups were considered statistically significant for *p* < 0.05.

## 4. Conclusions

In conclusion, we revealed that the MLCK expression was higher in the livers of HCC rats than in normal and resveratrol-treated rats. High level of MLCK expression was associated with inducing liver tumorigenesis and anti-apoptotic effects. Resveratrol down-regulated MLCK expression and inhibited HCC tumorigenesis via inducing apoptosis in tumor cells using a rat model of DENA-induced HCC.

## Figures and Tables

**Table 1:** Body and liver weights of different groups of rats (mean ± S.D.).

Group	*n*	Body weight (g)	Liver weight (g)	Liver weight (g)/Body weight (g) × 100%
A	8	309.4 ± 23.2	10.12 ± 2.01	3.26 ± 0.23
B	8	307.9 ± 32.1	10.03 ± 2.89	3.25 ± 0.31
C	8	278.3 ± 31.2	13.08 ± 3.24	4.69 ± 0.47 **
D	8	280.1 ± 29.6	14.02 ± 3.53	4.71 ± 0.56 **
E	8	299.3 ± 27.1	11.82 ± 2.25	3.91 ± 0.36 *

** *p* < 0.01 compared with group A;

* *p* < 0.05 compared with groups A and C.

**Table 2:** Effect of resveratrol supplementation (50 mg/kg body weight/day) on the nodule incidence % and the development of persistent nodules in the livers of rats with diethylnitrosamine (DENA)-induced hepatocellular carcinoma (HCC) (mean ± S.D.).

Group	*n*	Number of rats with nodules	Nodule incidence %	Total number of nodules	Average number of nodules per nodule-bearing liver
C	8	8	100%	297	36.8 ± 4.3
D	8	8	100%	291	36.2 ± 6.9
E	8	5	62.5% *	53	10.1 ± 2.6 **

* *p* < 0.05 compared with group C (Fisher’s exact probability test);

** *p* < 0.01 compared with group C (Student’s *t*-test). Use student’s *t*-test in Section 3.7.
